# *N*-Myristoyltransferase Inhibition in Parasitic Pathogens: Insights from Computer-Aided Drug Design

**DOI:** 10.3390/molecules30183703

**Published:** 2025-09-11

**Authors:** Fernanda de França Genuíno Ramos Campos, Willian Charles da Silva Moura, Diego Romário-Silva, Rodrigo Santos Aquino de Araújo, Inês Morais, Sofia Cortes, Fátima Nogueira, Ricardo Olimpio de Moura, Igor José dos Santos Nascimento

**Affiliations:** 1 Drug Development and Synthesis Laboratory, Department of Pharmacy, State University of Paraíba, Campina Grande 58429-500, Brazil; fernanda.campos@aluno.uepb.edu.br (F.d.F.G.R.C.); williancsmoura@gmail.com (W.C.d.S.M.);; 2 Department of Dentistry, State University of Paraíba, Campina Grande 58429-500, Brazil; diegoromarioo@gmail.com; 3 Postgraduate Program in Natural and Bioactive Synthetic Products, Federal University of Paraíba, João Pessoa 58051-900, Brazil; rodrigobiologojp@gmail.com; 4 Postgraduate Program of Pharmaceutical Sciences, Pharmacy Department, State University of Paraíba, Campina Grande 58429-500, Brazil; 5 Global Health and Tropical Medicine (GHTM), Associate Laboratory in Translation and Innovation Towards Global Health (LA-REAL), Instituto de Higiene e Medicina Tropical (IHMT), Universidade NOVA de Lisboa (UNL), Rua da Junqueira 100, 1349-008 Lisboa, Portugal; ines.morais@ihmt.unl.pt (I.M.); scortes@ihmt.unl.pt (S.C.);

**Keywords:** leishmaniasis, human African trypanosomiasis, malaria, myristoyl-CoA, NMT inhibitors, parasitic diseases

## Abstract

Neglected tropical diseases (NTDs) constitute a group of infectious diseases that severely affect the health of impoverished populations, and the health, economies, and health systems of affected countries. Leishmaniasis and human African trypanosomiasis (HAT) are particularly notable, and malaria, despite not being neglected, is part of the “big three” (HIV, tuberculosis, and malaria) with high incidence, increasing the probability of infection by NTDs. Therefore, efforts are ongoing in the search for new drugs targeting the enzyme *N*-myristoyltransferase (NMT), a potential drug target that has been explored. Thus, we provide a review here that highlights the epidemiological data for these diseases and the importance of discovering new drugs against these agents. Here, the importance of NMT and its inhibitors is clear, with this study highlighting thiochromene, pyrazole, thienopyridine, oxadiazole, benzothiophene, and quinoline scaffolds, identified by computational methods followed by biological assays to validate the findings; for example, this study shows the action of the aminoacylpyrrolidine derivative **13** against *Leishmania donovani* NMT (IC_50_ of 1.6 nM) and the pyrazole analog **23** against *Plasmodium vivax* NMT (IC_50_ of 9.48 nM), providing several insights that can be used in drug design in further work. Furthermore, the selectivity and improvement in activity are related to interactions with the residues Val^81^, Phe^90^, Tyr^217^, Tyr^326^, Tyr^345^, and Met^420^ for leishmaniasis (L*m*NMT); Tyr^211^, Leu^410^, and Ser^319^ for malaria (P*v*NMT); and Lys^25^ and Lys^389^ for HAT (T*b*NMT). We hope our work provides valuable insights that research groups worldwide can use to search for innovative drugs to combat these diseases.

## 1. Introduction

Neglected tropical diseases (NTDs) are a diverse group of conditions caused by various pathogens and associated with severe health, social, and economic deficits, primarily affecting the most impoverished communities, usually in tropical and subtropical areas [1,2]. They involve complex epidemiology, often intricately related to socioecological systems, environmental conditions, and complex life cycles [3,4,5].

The World Health Organization (WHO) currently registers the following conditions as NTDs: human African trypanosomiasis (HAT); leishmaniasis; Chagas disease (CD); schistosomiasis; dengue and chikungunya; rabies; mycetoma, chromoblastomycosis, and other deep fungal infections; leprosy; Buruli ulcer; trachoma; yaws; noma; onchocerciasis; dracunculiasis; echinococcosis; taeniasis/cysticercosis; foodborne trematodes; soil-transmitted helminthiases; lymphatic filariasis; ectoparasites; and snakebite envenoming [4,6,7]. Moreover, malaria is not considered an NTD, but it is a disease that affects tropical countries, mainly in Latin America and Africa, and is considered a public health problem [8,9,10]. Thus, malaria is included in the classification of “other diseases” along with Acquired Immune Deficiency Syndrome (AIDS) and tuberculosis. According to the Millennium Development Goals (MDGs), as with NTDs, research efforts and public policies are designed to end these epidemics [11].

Furthermore, some diseases have been subjected to thorough eradication campaigns, while others, such as vector-borne diseases, have a more widespread dissemination, making them challenging to combat, as climate changes favor the spread of insect vectors responsible for their transmission [12,13]. Among these, leishmaniasis, HAT, and malaria stand out due to high rates of morbidity and mortality, requiring more incentives in research and development of drugs to combat them, because the therapeutic arsenal is limited, with drugs that are difficult to administer and a constant rate of parasite resistance to current drugs, which inspires researchers to search for new drugs that focus on new mechanisms of action to overcome the limitations of current therapies [14,15].

The enzyme glycylpeptide *N*-tetradecanoyltransferase (EC:2.3.1.97), or *N*-myristoyltransferase (NMT), is responsible for catalyzing the myristoylation reaction, or the transfer of myristate from myristoyl-coenzyme A to the *N*-terminal residue of several different eukaryotic proteins. It is present in many organisms, such as humans, protozoa, viruses, and fungi [16,17,18]. Therefore, research has focused on developing antiparasitic, antimicrobial, antifungal, and antiviral drugs due to the importance of NMT for the survival of these organisms [19,20,21]. This includes leishmaniasis, HAT, and malaria, where several studies highlight NMT as an excellent drug target and potentially lead to the development of drugs against these diseases with an innovative mechanism of action. Furthermore, these studies are accompanied by computational screening, which can yield important insights into the planning and development of drugs against these diseases [16].

Computer-aided drug design (CADD)-based studies may involve ligand-based drug design (LBDD) approaches based on the knowledge of molecules that are active against a specific condition (i.e., quantitative structure–activity relationship—QSAR, pharmacophore modeling, ligand-based virtual screening), or structure-based drug design (SBDD), based on information about the target (i.e., molecular docking, molecular dynamics simulations, homology modeling) [22,23]. Public open-source databases archiving data on both of these aspects of CADD are also available to the global community [24,25], while ligand-based techniques now benefit from the widespread boom of artificial intelligence (AI) technology, capable of summarizing large quantities of data into new conclusions and offering novel perspectives for drug development [26,27].

Finally, this review aims to provide a comprehensive overview of leishmaniasis, HAT, and malaria, exploring the role of NMT in the infectious processes of these diseases, and describing its structure, functions, catalytic mechanism, and strategies in molecular modeling and CADD methods for drug design. Finally, here, we provide a review that identifies potential NMT inhibitors designed against some NTDs using computational methods, highlighting successful examples, validation processes, and prospects in computational drug discovery.

## 2. An Overview of Leishmaniasis, HAT, and Malaria

### 2.1. Leishmaniasis

More than 95 countries worldwide are endemic for leishmaniasis [28]. The etiological agents of this disease are protozoan parasites from the genus *Leishmania*, comprising more than 20 species with clinical relevance, which belong to the family *Trypanosomatidae* and order *Kinetoplastida* [29]. Over 70 species of sand flies have been proven vectors of *Leishmania* spp. [30]. The parasitic infection involves a diverse clinical spectrum reflecting the dissemination of *Leishmania* in macrophage-rich tissues [31].

This disease can be presented in three primary clinical forms: visceral leishmaniasis (VL), also known as kala-azar; cutaneous leishmaniasis (CL); and mucocutaneous leishmaniasis (MCL) [32]. Annually, 50,000–90,000 new cases of VL and up to one million cases of CL occur globally. VL is the most severe form, and if left untreated, the form can be fatal in more than 95% of cases [33]. The main symptoms of VL include irregular fever, weight loss, splenomegaly, hepatomegaly, and anemia. CL is the most common form and is characterized by benign, often self-healing skin lesions on exposed parts of the body. MCL causes partial or complete destruction of the mucous membranes in the nasal, oral, and pharyngeal cavities, which can lead to mutilating scars, causing stigma in affected persons [34].

In the absence of a human vaccine, control relies only on chemotherapy. First-line treatments include pentavalent antimonials (Sb^5+^), such as meglumine antimoniate and sodium stibogluconate (SSG), which demonstrate favorable clinical and microbiological outcomes in approximately 50% of patients but with side effects [35]. These compounds are believed to act by binding to polypeptides, inhibiting enzymes like DNA topoisomerase, and inducing alterations in the parasite’s plasma membrane. The liposomal form of amphotericin B presents high efficacy for visceral leishmaniasis and lower toxicity, but is expensive and requires intravenous administration. Miltefosine is the first effective oral drug used for both visceral and cutaneous forms, but raises concerns about teratogenicity and high cost. Despite their therapeutic potential, these treatments are associated with significant adverse effects, including local irritation, gastrointestinal disturbances, myalgia, arthralgia, elevated hepatic enzymes, and electrocardiographic abnormalities [36]. Furthermore, the increasing resistance of *Leishmania* to these drugs in recent decades underscores the urgent need to develop alternative therapies with novel mechanisms of action and reduced side effects [37,38].

### 2.2. Human African Trypanosomiasis (HAT)

Another parasitic disease of significant concern in tropical regions is HAT, which presents distinct epidemiological and therapeutic complexities [39]. This disease, also known as “sleeping sickness,” is caused by the protozoan parasites *Trypanosoma brucei gambiense* and *Trypanosoma brucei rhodesiense*, transmitted by infected tsetse flies (*Glossina* spp). *T. brucei gambiense* predominates in West and Central Africa, causing a slowly progressing disease, whereas *T. brucei rhodesiense*, endemic to East and Southern Africa, results in a more acute and severe form [40,41]. Over the past decades, the number of reported cases has declined significantly, with fewer than 1000 cases recorded in 2018 and an estimated 55 million people at risk between 2016 and 2020 [40].

The life cycle of *T. brucei* involves two hosts: the tsetse fly and humans. In the insect vector, the parasite undergoes proliferative and non-proliferative stages, culminating in the formation of metacyclic trypomastigotes in the salivary glands, which are ready to infect humans [42]. In the human host, the parasite multiplies in the blood, lymph, and subcutaneous tissue, producing symptoms such as fever, headache, and lymphadenopathy during the hemolymphatic (early) stage. Upon crossing the blood–brain barrier, the parasite invades the central nervous system (CNS), leading to neurological disturbances, behavioral changes, and disruption of the sleep cycle during the meningoencephalitis (late) stage. Without treatment, HAT is generally fatal [43,44].

The disease stage determines the treatment of HAT, which is assessed clinically and, in some cases, through cerebrospinal fluid analysis obtained via lumbar puncture [45]. Treatment strategies differ according to disease stage: in the early stage, pentamidine is effective against *T. brucei gambiense*, and suramin is effective against *T. brucei rhodesiense*. In the advanced stage, when the parasite crosses the blood–brain barrier, treatment becomes more complex. Although melarsoprol was historically used, its high toxicity has led to the adoption of the nifurtimox–eflornithine combination therapy (NECT) since 2009 [40,45]. Eflornithine (DFMO) inhibits the enzyme ornithine decarboxylase (ODC), which is essential for parasite proliferation, whereas nifurtimox induces oxidative stress within parasite cells. The combination therapy involves intravenous administration of eflornithine and oral administration of nifurtimox [45]. However, the toxicity, emergence of resistance, and administration challenges associated with these drugs underscore the need for novel therapies.

### 2.3. Malaria

*Plasmodium* is the protozoan responsible for malaria, with approximately 200 cataloged species, of which five infect humans: *P. falciparum*, *P. vivax*, *P. ovale*, *P. malariae*, and *P. knowlesi*. Among these, *P. falciparum* and *P. vivax* are responsible for the majority of cases [46]. Transmission occurs through the bite of an infected female *Anopheles* mosquito. *P. falciparum* predominates in sub-Saharan Africa, where malaria-associated mortality is highest, whereas *P. vivax* is more prevalent in the Americas. In 2023, there were 263 million cases and 597,000 deaths, with the vast majority reported in Africa [47].

Effective malaria treatment depends on three key factors: the patient’s clinical condition, the *Plasmodium* species responsible for the infection, and the geographical origin of the infection [48]. In specific regions such as Central America and Haiti, chloroquine remains effective [49,50,51]; however, artemisinin-based combination therapies (ACTs) are the gold standard, particularly for *P. falciparum*. ACTs combine short-half-life artemisinin derivatives, which rapidly reduce parasite load, with long-half-life partner drugs such as lumefantrine or amodiaquine [52]. This combination strategy ensures rapid reduction of parasitemia and clearance of residual parasites, thereby limiting the emergence of resistance and reducing treatment failure rates [48,53].

Nonetheless, the emergence and spread of ACT resistance, particularly in *P. falciparum*, represents an increasing challenge to malaria control. Artemisinin resistance, associated with mutations in the *Kelch13* (*K13*) protein, has been widely reported in Southeast Asia, particularly in the Greater Mekong Subregion [54,55]. More recently, validated *k13* mutations have also been detected in Africa, in Rwanda and Uganda, raising concerns about the geographical expansion of resistance [56,57]. Additionally, mutations in the *PfCRT* gene are linked to chloroquine resistance and have been implicated in the decreased sensitivity to partner drugs such as piperaquine [58,59].

These parasitic diseases remain major global health challenges. Although treatment has advanced, the rise of pathogen resistance highlights the urgent need for new therapies. Understanding the molecular mechanisms of pathogenesis and resistance is crucial for developing effective drugs and improving outcomes. Identifying and validating innovative therapeutic targets is critical to overcoming current limitations in antiprotozoal treatments. Additionally, there is a need to explore new mechanisms of action that can overcome the limitations of current therapies.

## 3. Targeting NMT: Insights in Drug Design

It is necessary to explore the discovery of drugs targeting new mechanisms of action in the search for an innovative product to overcome the limitations of current therapies against the diseases mentioned above [60]. In this way, interfering in the myristoylation process of proteins may be promising in the development of new agents. *N*-terminal myristoylation of proteins is crucial, both during and after protein translation. It plays a crucial role in regulating the interaction of these proteins with cell membranes, thereby influencing their localization and function. Therefore, adding myristoyl groups to the *N*-terminal glycine residue facilitates the association of proteins with the lipid membrane, where they often exert essential functions [20]. This process is catalyzed by NMT, which belongs to the GCN5 acetyltransferase superfamily, and catalyzes the myristate group of myristoyl-coenzyme A (myristoyl-CoA) to the *N*-terminus or internal glycine residue of a protein, forming a covalent bond [19]. NMT is present in several eukaryotic organisms [20], including parasites such as the protozoa *P. falciparum* [42], *L. Major* [61], *L. donovani* [62], *Trypanosoma brucei* [63], and others. Therefore, the following sections will describe the structure and functions of NMT and its application in drug design.

### 3.1. Structure and Functions of NMT

Protein *N*-myristoylation involves the addition of the 14-carbon saturated fatty acid, called myristate, to the glycine residue at the *N*-terminus of specific cellular proteins [19]. This reaction is catalyzed by the enzyme myristoyl-CoA: protein *N-*myristoyltransferase (NMT). Adding the myristoyl group to the protein affects its interaction with cell membranes, subcellular localization, and, consequently, biological function [64]. Specifically, the acceptor group of the amino-terminal region undergoes dynamic interactions around a catalyst platform centered on residue Thr^282^ [65]. In the case of humans, there are two main known NMT isoenzymes: H*s*NMT1 (Homo sapiens *N*-myristoyltransferase 1), which is formed by 496 amino acid residues [66], and H*s*NMT2 [67], often expressed in some diseases such as cancer and parasitic infections [20,68]. *N*-myristoylation is a critical process in several organisms, serving as a lipid modification of proteins to direct them to the membrane surface [69,70]. Then, it can facilitate interactions between proteins, enhance protein–membrane interactions, and alter protein stability [71]. Hence, *N*-myristoylation and consequent lipidation are critical to protein stability and membrane partitioning in several organisms, including protozoa and parasites [72]. In this way, it is critical in the drug design process to focus on selectivity, and several works highlight the patterns of target selectivity to discover a promising drug with this mechanism. For example, high-affinity inhibitors of P*v*NMT (*P. vivax N-*myristoyltransferase) provide critical π-π stacking interactions with Tyr^211^ and Tyr^334^, as well as hydrogen bonding with Asn^365^ and His^213^. Then, these features can be explored to design a more selective drug compared with human NMTs [73]. Ultimately, this information can be utilized to design new drugs through various methods, such as structure-based drug design (SBDD), thereby providing innovative therapeutic options.

### 3.2. Structure-Based Drug Design (SBDD) to Discover NMT Inhibitors

**Figure 1:**
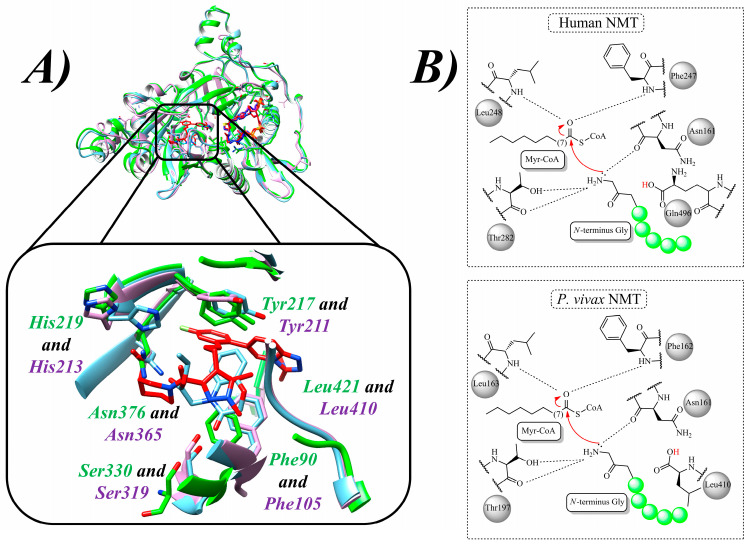
Comparison of several NMTs and Myr-CoA catalysis: (**A**) 3D structures of the NMTs from *L. donovani* in green *(PDB id:* 2WUU), *L. major* in cyan (PDB id: 5AG7), and *P. vivax* in magenta (PDB id: 5V0X), highlighting the critical residues Phe^90^, Tyr^217^, His^219^, Asn^376^, and Ser^330^ (*L. donovani*), and Phe^105^, Tyr^211^, His^213^, Asn^365^, and Ser^319^ (*P. vivax*); and (**B**) general *N*-myristoylation mechanisms in H*s*NMT and P*v*NMT.

Due to the availability of the X-ray structure of the parasitic NMT, several works use it to design inhibitors and provide insights into drug design [62]. Then, it was found that His^219^ (His^213^ in P*v*NMT) and Ser^330^ (Ser^319^ in P*v*NMT) (Figure 1A) are critical in the binding modes of the inhibitors and can be explored to develop the most selective drugs [74]. Furthermore, Asn^376^ (Asn^365^ in P*v*NMT) (Figure 1A) is related to the ligand positioning depending on the linked group [75], and Tyr^217^ (Tyr^211^ in P*v*NMT) (Figure 1A) is critical to the binding of the ligands [76]. Thus, some works show that interactions with Tyr^211^ (Tyr^217^ in L*m*NMT) and Tyr^334^ (Tyr^345^ in L*m*NMT) (Figure 1A) are related to the positioning of the ligand to provide an H-bond with His^213^ and Asn^376^ [73,77,78,79]. Finally, interactions with Phe^90^ (Phe^105^ in P*v*NMT) are crucial to offer highly selective inhibitors [80]. In addition, previous works highlight the importance of *C*-terminus Leu^410^ (Leu^421^ in L*m*NMT) (Figure 1A) in the selectivity of the ligands [81]. Finally, the variant *P. falciparum* G386E results in resistance to the inhibitors and continuous myristoylation, and this residue can be explored using CADD methods to overcome the resistance [82].

Furthermore, regarding substrate selectivity compared to H*s*NMT and P*v*NMT, crystallographic analysis of Myr-CoA binding revealed that the *C*-terminal residue Leu^410^ in P*v*NMT abstracts a proton from the *N*-terminal, whereas in H*s*NMT, it is Gly^496^ that does so (Figure 1B). Interestingly, this can be explored to improve target selectivity. Continuing the mechanism, Thr^197^ (Thr^282^ in H*s*NMT) and Asn^161^ contribute to stabilizing the amino group, thereby facilitating its nucleophilic attack on the carbonyl of the Myr-CoA thioester. The resulting tetrahedral intermediate is stabilized within the oxyanion hole, formed by Phe^162^ and Leu^163^ (Phe^247^ and Leu^248^ in H*s*NMT) (Figure 1B). As the reaction proceeds, CoA is released, followed by the formation and release of the peptide product [82]. Conversely, analysis of the binding modes of high-affinity inhibitors of P*v*NMT revealed critical π-π stacking interactions with Tyr^211^ and Tyr^334^ residues, along with hydrogen bonding involving His^213^ and Asn^365^ [73]. Thus, owing to the distinct substrate binding specificities involved in the *N*-myristoylation process between humans and parasites, it is possible to design highly selective inhibitors, reinforcing the potential of NMT as a promising antiparasitic drug target [83].

## 4. NMT Inhibitors Against Parasitic Diseases Identified by Computational Methods

The importance of NMT in designing new drugs against parasitic diseases is highlighted in several works. In addition, it is clear that computational methods are essential in drug design and any drug design campaign [84,85]. Hence, the following topics will describe the main NMT inhibitors discovered by computational methods and their implications in drug design and development against NTDs and related diseases, focusing on leishmaniasis, HAT, and malaria.

### 4.1. NMT Inhibitors Against Leishmaniasis

#### 4.1.1. Chromone Analogs

**Figure 2:**
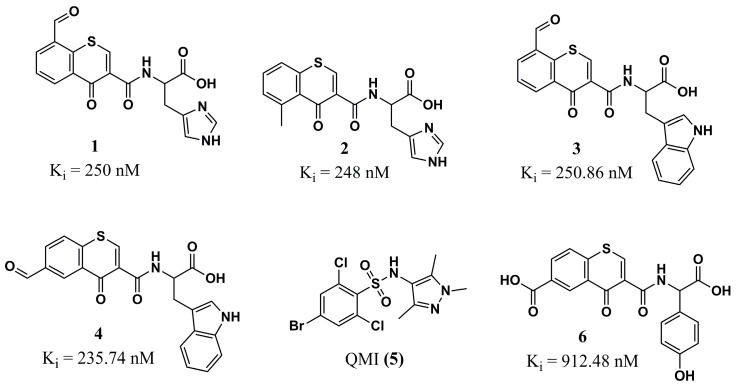
Chemical structures of cocrystallized ligand QMI **(5)** and chromone analogs **1**, **2**, **3**, **4**, and **6** from Johri et al. (2023) [86].

Johri et al. (2023) [86], based on the studies of Ribeiro et al. (2015) [87], in which molecular docking was carried out on a variety of flavonoid compounds in *Leishmania major N*-myristoyltransferase (L*m*NMT, PDB ID: 4A30), identified the chromone portion of these compounds as an essential constituent for their anti-*Leishmania* activities, by inhibiting protein myristoylation. This scaffold was therefore used as a starting point for designing a series of thiochromone derivatives substituted with various functional groups and anchored with aromatic amino acids, resulting in a total of 128 ligands to be analyzed by molecular docking (Glide and Auto-Dock) against NMT from *L. major*. In silico post-docking analyses and ADMET pharmacokinetic predictions allowed the selection of eight promising *hit* compounds. The prediction of the *Lm*NMT inhibition constant (K_i_) highlighted the derivatives **1**, **2**, **3**, and **4** (Figure 2) as the most promising. Curiously, these compounds show two amino acids with heterocyclic side chains (histidine and tryptophan), revealing their importance, in addition to the essential role of the presence of electron-withdrawing (aldehyde) groups in positions 8 and 6 for compounds **1**, **3**, and **4**, and a moderate electron-donating group in position 5 for compound **2** (methyl).

In a complementary way, MD simulations (RMSD, RMSF, and R_g_) were carried out in comparison with the cocrystallized ligand QMI **(5)** (Figure 2), which again highlighted the promising potential of derivatives anchored to the amino acid tryptophan, highlighting compound **3**, which provides the best stability at the binding site of the L*m*NMT. However, they also indicate promising characteristics for compounds related to the amino acid tyrosine, especially compound **6** (Figure 2), evidenced by the importance of further work with derivatives linked to these amino acids. Two-dimensional studies reveal significant interactions between these compounds and the cocrystallized ligand, specifically with the L*m*NMT amino acid residues Tyr^217^ and Phe^90^, as well as demonstrating that compound **3** exhibits the highest number of interactions with the target [86]. Finally, this work provides critical insights into computational chemistry that can be used in further optimization of this chemical scaffold, with a great perspective to evaluate in experimental assays.

#### 4.1.2. Peptidomimetics

**Figure 3:**
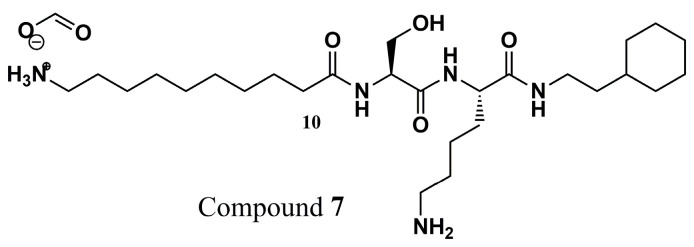
Chemical structure of peptidomimetic compound **7** from Olaleye and colleagues (2014) [88].

Since peptide structures represent NMT substrates, Olaleye and colleagues (2014) [88] synthesized a series of peptidomimetic compounds to evaluate their critical interactions and propose NMT as the main mechanism of action. Thereby, it was found that there were significant interactions between compound **7** (Figure 3) and L*m*NMT, similar to the cocrystallized complexes. Additional kinetic studies confirm the action of this peptidomimetic derivative as a competitive inhibitor of *Leishmania donovani* NMT (*Ld*NMT), an enzyme 97% identical to *Lm*NMT. Unfortunately, the inhibitory potential studies also showed selectivity for the H*s*NMT (IC_50_ of 0.024 ± 0.003 µM for *Ld*NMT and IC_50_ of 0.06 ± 0.003 µM for *Hs*NMT). Despite this, the binding modes of compound **7** in P*v*NMT are critical, mainly related to the electrostatic interactions of the *N*-terminus with Leu^410^ and the hydroxyl with His^213^, with a similar binding mode in the L*m*NMT, and are comparable due to the high similarity of the two enzymes. Finally, these key insights into experimental assays and correlation with their binding modes can be helpful in the rational design of promising new derivatives.

#### 4.1.3. Thienopyrimidine, Piperidinylindole, and Aminoacylpyrrolidine

**Figure 4:**
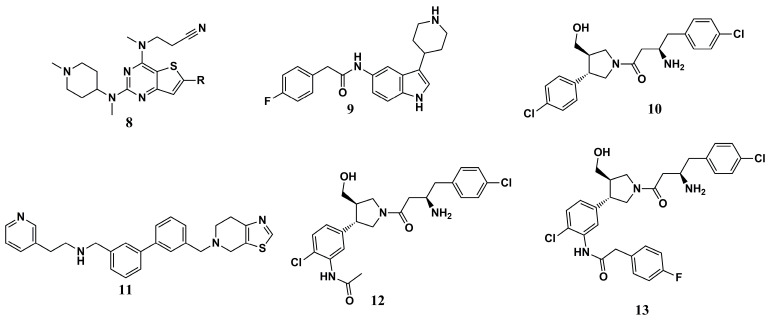
Chemical structures of compounds **8**, **9**, **10**, **11**, **12**, and **13**, analogs of thienopyrimidine, piperidinylindole, and aminoacylpyrrolidine identified by Brannigan et al. (2014) [80] and Hutton et al. (2014) [90].

From a set of Pfizer global structures, Bell et al. (2012) [89] performed an HTS of 150,000 molecules, allowing the selection of submicromolar *Ld*NMT inhibitors representing the classes of thienopyrimidine **(8)**, piperidinylindole **(9)**, aminoacylpyrrolidine **(10)**, and biphenyl **(11)** (Figure 4), and identified compounds **9** and **10** as the most promising. Based on these promising data, Brannigan et al. (2014) [80] evaluated the intensity and binding mode against L*m*NMT, which shares 97% amino acid sequence identity with the same protein from *L. donovani*.

An analysis of the mode of interaction in a cocrystallized complex between *Lm*NMT, the substrate myristoyl-CoA, and compound **9** (IC_50_ of 0.318 µM and 200-fold selectivity for *Hs*NMT) demonstrated the critical binding mode in the peptide-binding region in the interaction with its *C*-terminal portion. The structure of compound **9** has been shown to interact with the side chain of the Phe^90^ residue. In addition, the possibility of a dipole–dipole interaction between the fluorine substituent of compound **9** and the acid sequence Glu^82^-Asp-Asp-Asp^85^, as well as the visualization of the formation of a series of polar interactions and H-bond formation sites involving the atoms present in the amide group of the ligand, stand out.

Furthermore, the best *Ld*NMT inhibitory value was obtained for compound **10** (IC_50_ of 0.077 µM, with 80-fold selectivity for *Hs*NMT), which demonstrated apolar interactions of one of its chlorophenyl rings with the Val^81^ and Phe^90^ residues. In addition, polar interactions were observed between the hydroxyl group of the ligand and the Tyr^326^, Leu^421^, and Met^420^ residues. The amine and carbonyl groups of the ligand facilitated the formation of various polar interactions and hydrogen-bonding points with essential residues of the molecular target. For both ligands, polar interactions maintained with Leu^421^ seem to be responsible for maintaining their inhibitory potential, as do interactions with Tyr^217^, which represents a point of differentiation from *Hs*NMT and may be essential in the inhibitory selectivity to the parasite [80].

In another work, a Pfizer corporate collection was used as a source of molecules for an HTS in search of new *Ld*NMT inhibitors. In this way, four promising series were identified, with proof of their potential against *Ld*NMT and *Lm*NMT for structures **9** and **10** [89]. These compounds inspired the development of hybrid derivatives **12** and **13** (Figure 4) by Hutton et al. (2014) [90]. The *para*-fluorophenyl moiety in compound **13** resulted in a very low IC_50_ in the biochemical assay and an *Ld*NMT inhibition constant value of 1.6 nM. Examination of the binding mode revealed that this derivative preserves the interactions of both **9** and **10** with L*m*NMT, specifically the peptide-binding portion of the enzyme, which corroborates its superior *Ld*NMT inhibition values. In addition, critical interaction with Thr^203^ by the hydroxy group can be related to the potential of these compounds. Unfortunately, this improvement was not reflected in better EC_50_ values in tests with *L. donovani* amastigotes, but these findings could be critical for using this chemical scaffold to design new analogs with improved activity.

#### 4.1.4. Pyran-Acrylate Analogs

**Figure 5:**
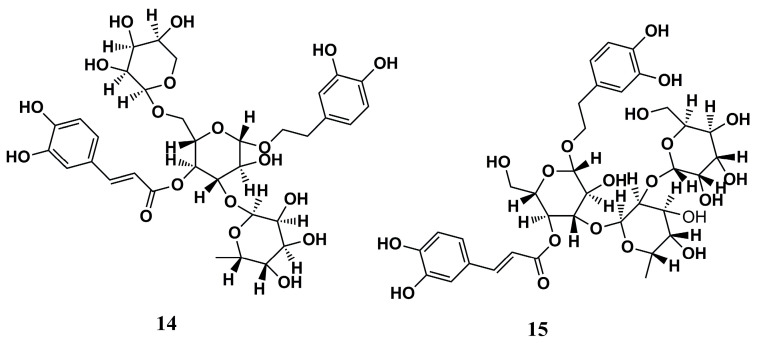
Chemical structures of compounds **14** and **15**, pyran-acrylate analogs identified by García-Sosa (2018) [91].

The chemical library ZINC database, along with a set of other biologically active structures, was used by García-Sosa (2018) to provide compounds for evaluating target binding affinity by molecular docking against L*m*NMT (PDB ID: 4A30) [91]. Among the structures evaluated, compounds **14** and **15** (Figure 5) stood out for their great docking scores (−13.93 and −14.75 kcal/mol, respectively) and binding energies (−102.46 and −83.01 kcal/mol, respectively) and for not being predicted as inhibitors of the H*s*NMT, demonstrating that they can be utilized as good *hits* in the development of new NMT inhibitors against *Leishmania* sp.

#### 4.1.5. Pyrrole Analogs

**Figure 6:**
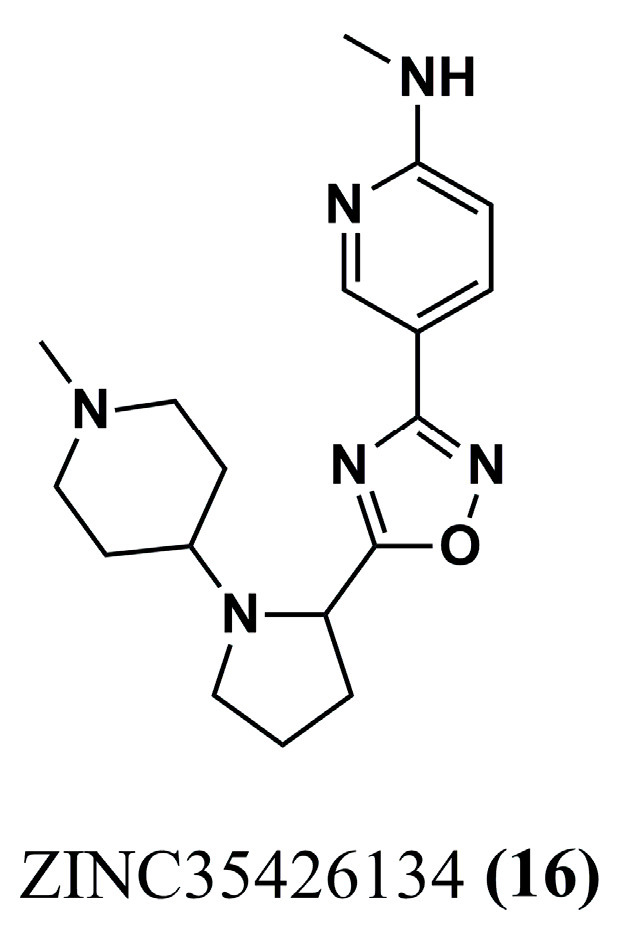
Chemical structure of ZINC35426134 **(16)**, pyrrole analog identified by de Carvalho Gallo et al. (2018) [92].

*Leishmania braziliensis N*-myristoyltransferase (*Lb*NMT) inhibitors were initially selected through a hierarchical virtual screening approach to construct a pharmacophoric model (Galahad^®^ module of SYBYL-X 2.0) based on physicochemical and structural characteristics. The best model selected had the following pharmacophoric characteristics: four hydrophobic centers, four H-bond acceptors, and a positive nitrogen center, which is compatible with *Lb*NMT ligand–receptor (PDB ID: 5A27) interactions. Therefore, using ligand-based and structure-based virtual screening strategies, de Carvalho Gallo et al. (2018) [92] identified six molecules that were compatible with the pharmacophore model and were subsequently selected for molecular docking against L*m*NMT (PDB ID: 5A27), which has 100% identity with the binding site of L*b*NMT. Of this series of six molecules, ZINC35426134 **(16)** (Figure 6) showed the best intermolecular interaction related to the pharmacophore model and the best GRID score of −63.87 kcal/mol (DOCK 6.8 software). These data suggest an excellent compound that can be explored in further work.

The structural features of ZINC35426134 **(16)** enabled hydrophobic interactions with the Phe^82^, Tyr^209^, Val^370^, and Leu^391^ residues while also demonstrating hydrogen bond formation with Asn^159^, Tyr^318^, and Val^370^. Next, to identify more critical information about the binding of the ligands, MD simulations (GROMACS) were performed, and a hydrophobic interaction site was observed between the aromatic nitrogen heterocycle within the molecule and Tyr^345^, while H-bond formation involved the Tyr^217^ and Leu^421^ residues. These sites and types of interaction appear to be starting points for elucidating the mechanism of action and rational design of other potent NMT inhibitors of *Leishmania* sp. [92].

#### 4.1.6. Natural Compounds

**Figure 7:**
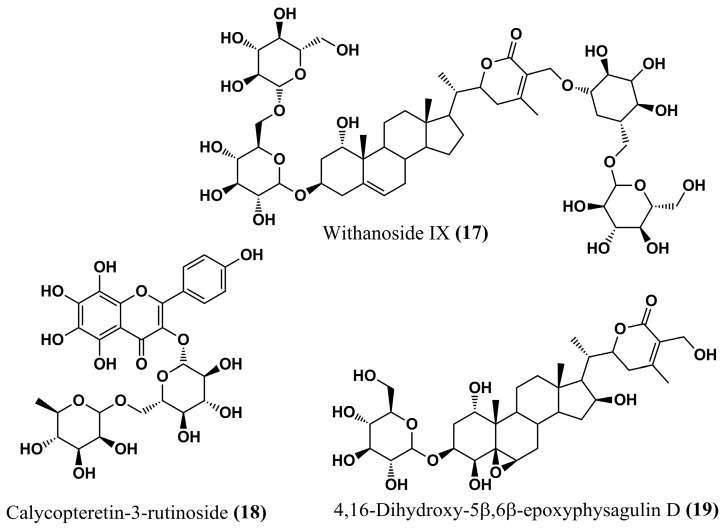
Chemical structures of withanoside IX **(17)**, calycopteretin-3-rutinoside **(18)**, and 4,16-dihydroxy-5β,6β-epoxyphysagulin D **(19)** isolated from *Withania somnifera (L.) Dunal* by Orabi et al. (2023) [93].

A series of natural isolates obtained from *Withania somnifera (L.) Dunal* was analyzed according to its anti-*Leishmania* potential by Orabi et al. (2023) [93] through molecular docking (PyRx software) and MD simulations (NAMD 3.0 software) against *Lm*NMT. A total of 167 compounds were initially screened by docking, and the best compounds were evaluated by MD simulations through stability of the complex with the enzyme, as assessed by Root Mean Square Deviation (RMSD), leading to the selection of 10 candidates with the highest affinity indices to the target. Then, the compounds withanoside IX **(17)**, calycopteretin-3-rutinoside **(18)**, and 4,16-dihydroxy-5β,6β-epoxyphysagulin D **(19)** (Figure 7) showed outstanding potency, with binding affinity values better than −22.0 kcal/mol, indicating that they can be promising in further assays.

All three compounds formed hydrogen bonds with the Met^420^ residue of L*m*NMT, highlighting a potentially critical site for inhibitory activity. In addition, calycopteretin-3-rutinoside **(18)** showed H-bond formation with Leu^421^, a vital contact amino acid of the NMT active site. The great in silico results for these three compounds were corroborated by their good binding stability to the NMT binding site, which was evaluated via MD simulation studies. The encouraging anti-Leishmania activities of *W. somnifera* extracts suggest that these promising candidates in in silico studies may represent viable alternatives in the search for new potent drugs against this parasite or even inform the rational design of new promising compounds [93].

Some data in the literature acknowledge the difficulty in designing new compounds to inhibit the NMT of *Leishmania* sp., and they attribute this to a lower susceptibility to inhibiting this target compared to other organisms [94,95]. However, knowledge of the structure of the NMT molecular target of *Leishmania* sp., especially the binding site of its substrates, is a strong ally in the search for anti-*Leishmania* drugs, strengthened by the help of computer-aided drug design tools. Brannigan et al. (2010) [62], for example, describe, among the NMT contact amino acids, residues such as Val^81^, Phe^90^, Tyr^217^, Tyr^326^, Tyr^345^, Met^420^, and Leu^421^, at least one of which has been shown to interact with the structures described in this section, which could guide the pathway towards understanding the inhibitory mechanism of action of new drugs. Finally, these features can help researchers to discover promising NMT inhibitors against leishmaniasis.

### 4.2. NMT Inhibitors Against Plasmodium sp.

#### 4.2.1. Pyrazole Analogs

**Figure 8:**
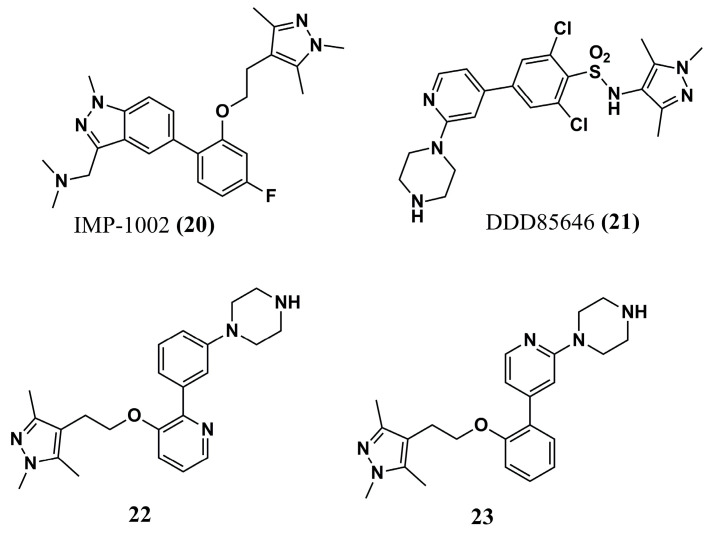
Chemical structures of pyrazole analogs IMP-1002 **(20)**, DDD85646 **(21)**, **22**, and **23** identified by Rodríguez-Hernández et al. (2023) [96].

Rodríguez-Hernández et al. (2023) [96] developed a series of hybrids to obtain potent, selective candidates for P*v*NMT and understand the molecular basis of its selectivity. The design of the new hybrids was motivated by the well-known *Pv*NMT inhibitors IMP-1002 **(20)** and DDD85646 **(21)** (Figure 8), which target the Tyr^211^, Ser^319^, and Leu^410^ residues. Of the four series obtained, compound **22** (IC_50_ of 80.15 nM for *Pv*NMT and Selectivity Index—SI of 259.2) and compound **23** (IC_50_ of 9.48 nM and SI of 63.1) (Figure 8) stand out for their potency and high selectivity. Next, compound **22** was selected for cocrystallization with *Pv*NMT and the MyrCoA substrate for further molecular interaction studies.

These X-ray studies demonstrated interaction with a hydrophobic pocket in the peptide-binding region, which contains both the *N*- and *C*-terminal domains. In comparison with the precursors IMP-1002 **(20)** and DDD85646 **(21)**, the structure of compound **23** was stabilized in a rotated Tyr^211^ conformation, which determined a state of selectivity in the interaction with IMP-1002 **(20)**. Additionally, its 1,3,5-trimethylpyrazole portion led to an H-bond with Ser^319^. Finally, compound **23** was a promising inhibitor of the liver stage of *P. vivax* (2.3–4.6 µM against schizonts and 1.7 µM against hypnozoites), representing a critical scaffold that can be explored in further works of optimization.

#### 4.2.2. Piperidine Analogs

**Figure 9:**
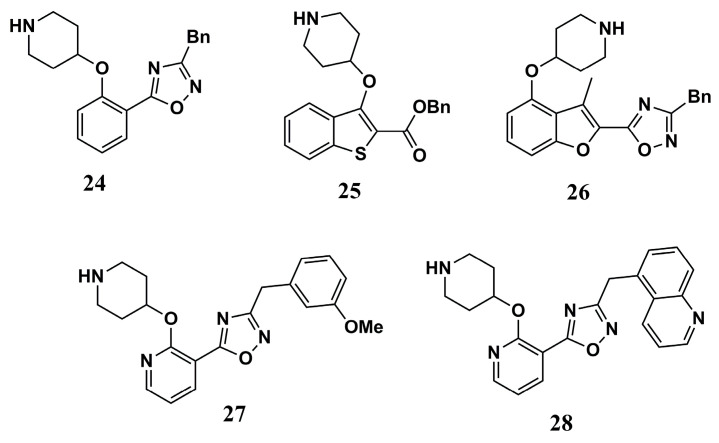
Chemical structures of piperidine analogs **24**, **25**, **26**, **27**, and **28** identified by Yu et al. (2015) [97].

Compound **24** (Figure 9) was developed by molecular simplification and modification strategies from compounds **25** and **26** (Figure 9) and had K_i_ values against *P. falciparum N*-myristoyltransferase (*Pf*NMT) and a selectivity index comparable to these precursors (K_i_ of 1.4 µM in *Pf*NMT and 33 µM in *Hs*NMT). Modifications in compound **24** resulted in compound **27** (Figure 9), which is more potent against P*v*NMT, with a K_i_ value of 0.027 µM. However, it shows a K_i_ of 0.27 µM against *Hs*NMT inhibition. Next, the crystallography of compound **27** suggests that its increased potency is due to the formation of an additional hydrogen bond with Tyr^315^. Additionally, replacing the methoxyphenyl portion with quinolinyl further reduced the potency of compound **28** (K_i_ of 0.0017 µM for P*f*NMT) (Figure 9) while maintaining an SI of 14 [97]. These findings were critical to reveal insights in medicinal chemistry to design new promising inhibitors and the most selective drugs.

#### 4.2.3. Piperazine, Steroid, and Thiazolidine Derivatives

**Figure 10:**
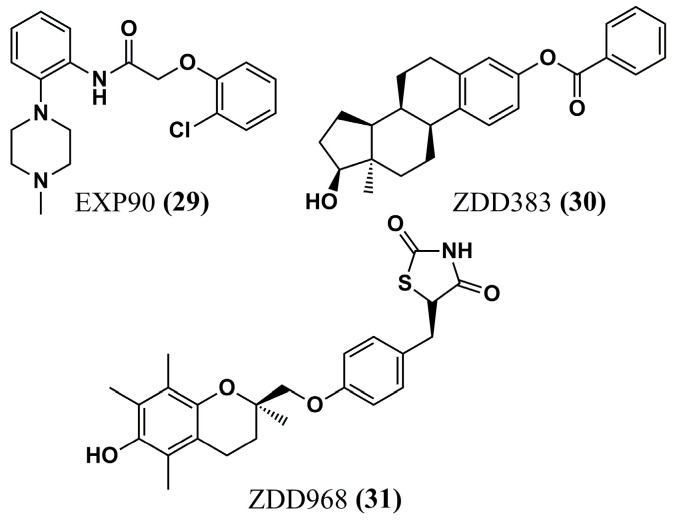
Chemical structures of EXP90 **(29)**, ZDD383 **(30)**, and ZDD968 **(31)**, piperazine, steroid, and thiazolidine derivatives, respectively, identified by Nicolau et al. (2023) [98].

Nicolau et al. (2023) [98] built models based on the shape of NMT inhibitors through ROCs (Receiver Operating Characteristics), one of which stood out for inhibitors that share six pharmacophoric characteristics. This model is based on a virtual screening to provide 500 molecules best ranked by the Tanimoto Combo score. Of these, the eight best-ranked compounds in the molecular docking (FRED program) analysis and subsequent MD simulation studies (GROMACS software) enabled the selection of ligands that formed the most stable complexes with the NMT protein: EXP90 **(29)**, ZDD383 **(30)**, and ZDD968 **(31)** (Figure 10).

Evaluation of the ligand–receptor interactions of the most stable complexes in MD simulations was carried out using LigPlot^+^ 2D, allowing us to observe that only ZDD698 **(30)** exhibits an interaction profile with P*v*NMT similar to known inhibitor benzofuran. Thus, the candidate showed π-π stacking with Phe^105^ and H-bond formation with Ser^319^, and its potential should be confirmed in future biological studies [98].

#### 4.2.4. Benzothiophene Analogs

**Figure 11:**
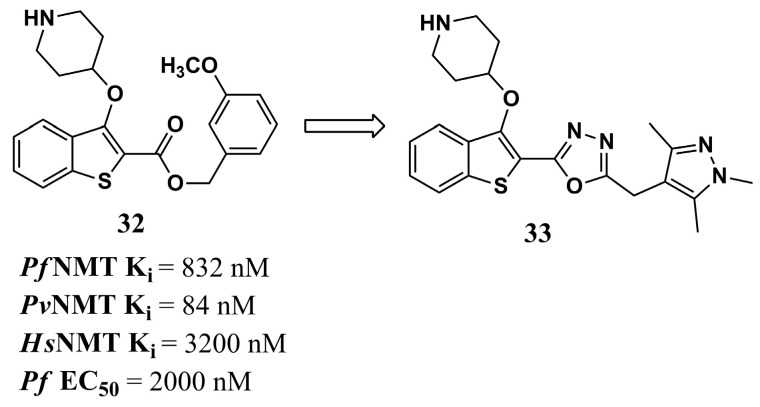
Chemical structures of benzothiophene compounds **32** and **33** from Rackham et al. (2014) [81].

Among the characteristics predicted to be important in the development of NMT inhibitors, ligand efficiency-dependent lipophilicity (LELP) has been shown to determine the drug-likeness profiles of these compounds, whose reduced values also tend to accompany a low toxicity profile [99]. Based on this parameter, Rackham et al. (2014) [81] were inspired by the structure of compound **32** (LELP of 13.5) (Figure 11), with crystallographic data published by Rackham et al. (2013) [100], leading to the conclusion that the insertion of a spacer between the ester and methoxyphenyl groups could improve the interaction profile with the *Pv*NMT target.

Hence, compound **33** (Figure 11) was planned and synthesized, and showed the best affinity values for *Pf*NMT (K_i_ of 8 nM) and *Pv*NMT (K_i_ of 2 nM), in addition to great antiparasitic potential against *P. falciparum* (EC_50_ value of 302 nM), followed by an excellent LELP value (5.5). However, it also showed an affinity for H*s*NMT. Even though the interaction between its pyrazole group and Ser^319^ did not occur as expected, according to the crystallographic data, there was an interaction by salt bridge with Leu^410^ and Tyr^107^, as well as interactions of the oxadiazole moiety between Phe^105^ and Tyr^211^. Although its potential in an in vitro cellular assay did not prove to be more efficient than the standard drugs chloroquine and atovaquone, and its EC_50_ values were similar against four strains of *P. falciparum* (3D7, NF54, K1 and Dd2) and liver-stage parasites (*P. berghei*), this highlights the conclusion that the structural modifications performed, directed by solubility parameters or points of interaction with the target, are an exciting method for future studies in the search for more potent and selective NMT inhibitors [81].

In another work, using QSAR also allowed the analysis of *Pf*NMT inhibitors, and their selectivity was compared to that of H*s*NMT in Garcia et al. (2022) [101]. The best inhibition model constructed made it possible to highlight the presence of the sulfur atom in the benzothiophene system, the oxadiazole ring, and the pyrazole nitrogen as important inhibitory and selective structural patterns of compound **33** (Figure 11). CoMFA contour maps were able to highlight that the N2 of the pyrazole ring may represent the point of differentiation in the higher affinity for *Pf*NMT than for *Hs*NMT. Additionally, bulky substituents in this ring’s 2-, 3-, and 5-positions indicate that they are favorable for an increased affinity to the parasite target. The oxadiazole group and the piperidine ring represent favorable regions for forming charge-mediated interactions with the drug target.

#### 4.2.5. Quinoline Analogs

**Figure 12:**
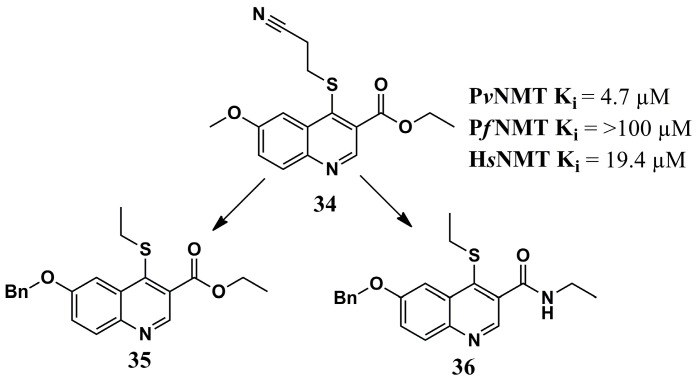
Chemical structures of quinoline analogs **34**, **35**, and **36** from Goncalves et al. (2017) [78].

Evaluation of the mode of interaction of compound **34** (Figure 12) in complex with *Pv*NMT and a substrate was used as the basis of investigation to obtain structural characteristics of the target by Goncalves et al. (2017) [78]. Quinoline derivative **34** occupies the hydrophobic pocket, interacting with Phe^103^ and Phe^105^ through π-π stacking. Additionally, an H-bond is formed between the nitrogen atom of this inhibitor and Ser^319^. Assessment of the interaction profile in this ternary complex allowed designing a series of modified quinoline derivatives to strengthen interactions with P*v*NMT. Of these, compounds **35** and **36** (Figure 12) provide the best results. Further, compound **36** reflected greater potency for both parasitic forms of NMT (K_i_ = 0.44 µM for *Pv*NMT and K_i_ = 0.67 µM for *Pf*NMT), maintained activity equivalent to compound **35** (K_i_ = 0.34 µM for *Pv*NMT and K_i_ = 0.96 µM for *Pf*NMT), and demonstrated better selectivity compared to its affinities against H*s*NMT (from 3-fold to >30-fold). Additional structural modifications enabled a significant increase in the inhibition of P*v*NMT and P*f*NMT while also enhancing the inhibitory potential for H*s*NMT. However, these data demonstrate how knowledge of the molecular target’s binding site can aid in constructing the chemical structure of a potential inhibitor.

In the next work, Goncalves et al. (2017) [78] inspired the design of quinoline-based P*v*NMT and P*f*NMT inhibitors by Jameel et al. (2023) [102], which continued the development of drugs based on the structure of the molecular target and the structural features of previously developed inhibitors. The modifications were primarily designed to enhance hydrogen-bonding interactions (with the nitrogen of the quinoline ring), increase electronic density in the aromatic system, and facilitate π-π stacking interactions (with the fused ring system).

**Figure 13:**
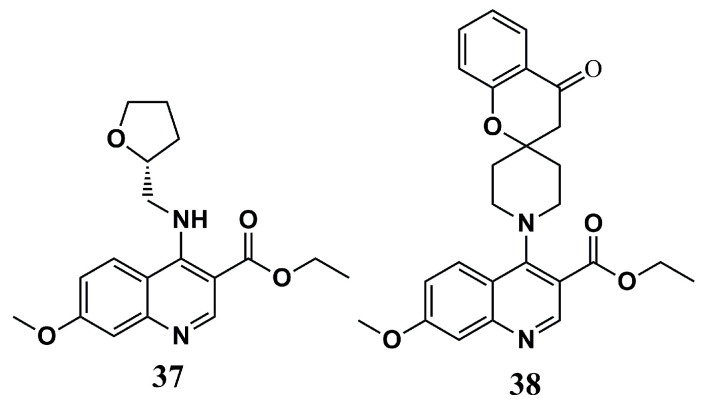
Chemical structures of quinoline derivatives **37** and **38** from Jameel et al. (2023) [102].

The molecular docking assays of Jameel et al. (2023) [102] demonstrated amino acid residues that were important in maintaining a solid interaction with the inhibitors, forming strong π-π stacking with P*v*NMT (Phe^105^, Tyr^211^, and Tyr^334^ residues) and P*f*NMT (Phe^105^, Tyr^211^, His^213^, and Phe^226^ residues), and hydrogen bonds with P*v*NMT (Tyr^334^ and Ser^319^ residues) and P*f*NMT (Ser^319^ and Ser^387^ residues). The maintenance of these interactions seemed to reflect the occupation at the same binding site of both targets evaluated, highlighting its fit with compounds **37** and **38** (Figure 13).

These compounds were evaluated according to their in vitro antimalarial activities against *P. falciparum* (*Pf*3D7) and *P. vivax* (*Pf*INDO). Both compounds proved to be potent against the two parasite strains, with IC_50_ values of 3.96 µM (*Pf*3D7) and 6.38 µM (*Pf*INDO) for compound **37** and 6.71 µM (*Pf*3D7) and 2.8 µM (*Pf*INDO) for compound **38**. Analysis of interference in the maturation stages of *Pf*3D7 also showed that these inhibitors can inhibit the parasite’s maturation. The in vitro hemolytic assay to determine toxicity in human host cells demonstrated a percentage of erythrocytic lysis below 10% for 25 µM of **37** and **38**, classifying them as non-toxic, providing critical information in drug design and a great chemical scaffold for further work [102].

#### 4.2.6. Oxadiazole Analogs

**Figure 14:**
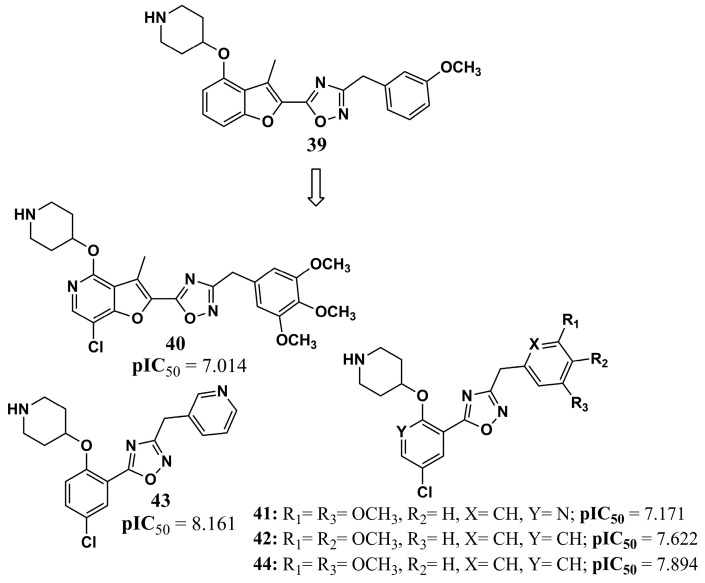
Chemical structures of oxadiazole **39** to **44** from Santos-Garcia et al. (2018) [103].

Santos-Garcia et al. (2018) [103] performed 4D-QSAR studies to search for new NMT inhibitors, where the evaluation of the models generated provides pharmacophoric analysis according to any non-polar interactions, charge densities, hydrogen bond formation points, and the existence of aromatic systems. The model identified the importance of the existence of these types of interactions for the great stability of the complex, in particular hydrogen bonds and the aromatic system, and therefore, compound **39** guided the design of compounds **40**, **41**, **42**, **43**, and **44** (Figure 14) against *Pf*NMT. The pIC_50_ values were better for all the structures obtained than for their precursor compound **39**, with a pIC_50_ value of 7.301. All the suggested structures had their pharmacokinetic parameters evaluated in silico, according to Lipinski’s Rule of Five, without any violation. Thus, these chemical structures and their physicochemical and pharmacokinetic properties may provide a basis for constructing inhibitors that prove to be potent against the NMT of *Plasmodium* sp.

These studies led us to observe amino acid residues that are important for maintaining interactions with the chemical structure of the inhibitor. The Tyr^211^ residue, for example, is recognized as responsible for the selectivity of action for the parasite’s NMT target to the detriment of its human form as a result of its conformational changes in each [97]. In addition, approximation and relationship to the Leu^410^ residue are also crucial for guiding the rational design of new drugs, as it plays an essential role in myristate transfer in *Plasmodium* NMT [82]. Interactions with Ser^319^ are observed in many of the results of the crystallographic studies cited in this section. In addition to elucidating the mode of binding to the protein site, these interactions can lead to an increase in the stability of the formed complexes, as well as binding to Tyr^211^ and Leu^410^.

### 4.3. NMT Inhibition Against Human African Trypanosomiasis (HAT)

#### 4.3.1. Pyrazole Analogs

**Figure 15:**
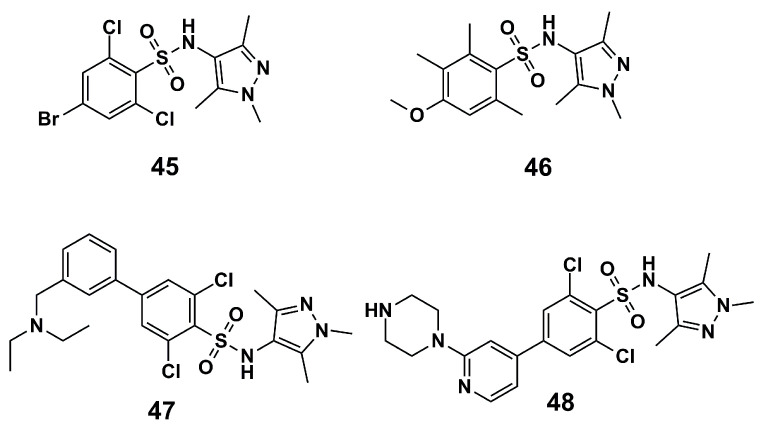
Compounds taken as models for *Tb*NMT inhibitory activity explanation [104].

Kumar et al. (2021) [104] have developed QSAR models for predicting *Trypanosoma brucei N*-myristoyltransferase (T*b*NMT) inhibitors based on 264 sulfonamides derived from the literature. Thereby, based on the most relevant structural attributes identified, the compounds were compared with the cocrystallized compound **45** (Figure 15) in *Lm*NMT. In this way, it was found that bromine at the *para* position and two chlorine atoms at the *ortho* position are responsible for a relative increase in *Tb*NMT inhibition compared to the other known inhibitor compound **46** (Figure 15). Additionally, the H-bonds of compound **45** are responsible for the optimal fit at the binding site. On the other hand, compound **47** (Figure 15), with a diethylamino-methyl group, provides high activity, as the presence of additional aromatic carbons, branched aromatic carbons, and nitrogen atoms increased activity compared to compound **45**, due to more π-π and σ-π interactions at the binding site. Finally, compound **48** (Figure 15) shows ethylamine fragments in the 3-piperazinyl-pyridine ring that increase activity compared to compound **47** by the addition of H-bonds. Accordingly, this work provides critical insights into drug design and compounds that may be promising for further optimization work.

In the next work, Sheng et al. (2009) [105] constructed 3D protein structures for P*f*NMT, L*m*NMT, and T*b*NMT using homology modeling and refined them using MD simulations. Then, known inhibitors of *Pf*NMT were docked, and their affinities were assessed. In this way, hydrophobic interactions and H-bonds were found to be mainly responsible for the binding. The selectivity over H*s*NMT was primarily attributed to H-bond interactions with Lys^25^ and Lys^389^. No H-bond interactions were observed with the enzyme’s *C*-terminal carboxylate, which is vital in its catalytic mechanism. Finally, the authors conclude that inhibitors must interact hydrophobically with two hydrophobic pockets to improve affinity against *Tb*NMT, forming hydrogen bonds to the *C*-terminal carboxylate and with residues in neutral, negatively charged, or positively charged hydrogen-bonding pockets.

In another work, Masand et al. (2019) [106] developed 2D-QSAR multilinear regression (MLR) models based on 270 pyrazole–sulphonamide inhibitors known to describe structural features to discover new drugs. Accordingly, the characteristics in favor of activity include H-bond acceptors at a bond distance from an oxygen atom and positively charged atoms at nine bonds from nitrogen atoms. In addition, the *N*-4-disubstituted-benzenesulfonamide group and its H-bond acceptor oxygen atoms, sulfur atoms, and H-bond acceptors within eight bonds, halogen and nitrogen atoms at a topological distance of seven, carbon and halogen atoms separated by a topological distance of nine, and others are shown to be critical to the binding of the inhibitor.

**Figure 16:**
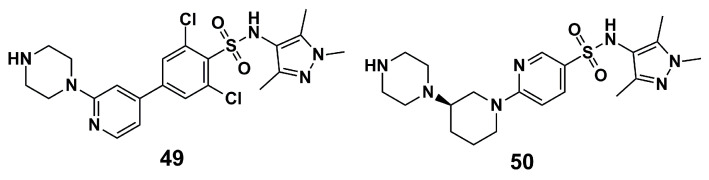
The most active *Tb*NMT inhibitors in the dataset subjected to metabolism prediction by Scotti et al. (2018) [107].

Similarly, Scotti et al. (2018) [107] applied molecular modeling to chemometric approaches in known pyrazole–sulphonamide T*b*NMT inhibitors, aiming to discover the structural characteristics responsible for inhibitory activity. A set of 62 inhibitors was subjected to energy minimization and geometry optimization by the AM1 semi-empirical quantum mechanics method. After excluding compounds with pIC_50_ values up to 4, 41 inhibitors remained. PCA analysis yielded 507 grid-independent descriptors obtained by searching for 3D intermolecular interactions using molecular interaction field (MIF) probes. Thus, after correlogram analysis of all descriptors, it emerged that lipophilicity, hydrophobic interactions, molecular shape, and H-bond accepting ability were essential for *Tb*NMT inhibition, and methyl groups in pyrazole rings contribute significantly. In the computational metabolism assays using the isoforms CYP1A2, CYP2C9, CYP2C19, CYP2D6, and CYP3A4, the most active compounds, **49** and **50** (Figure 16), demonstrate *N*-dealkylation to conceive their first metabolites, although the activity is not lost in these newfound molecules [107].

#### 4.3.2. Thiazolidin and Benzoxazine Analogs

**Figure 17:**
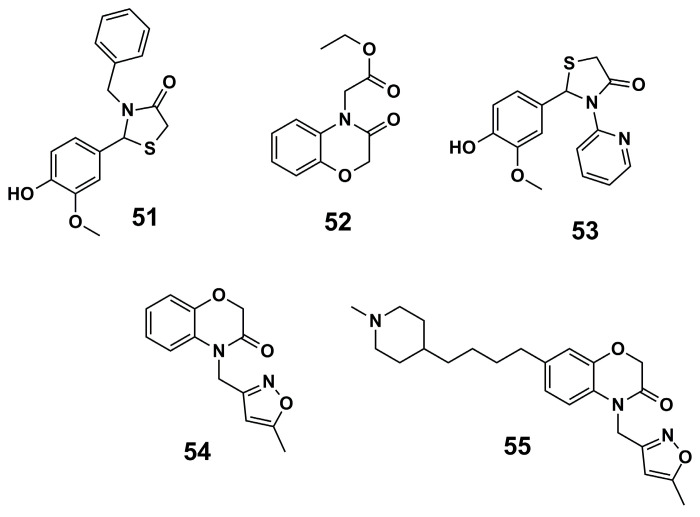
Analogs of thiazolidin and benzoxazine were identified as novel *Tb*NMT inhibitors [108].

Furthermore, a high-throughput virtual screening (vHTS) following a 3D pharmacophore search of the drug discovery unit Dundee *in-house* database was carried out by Spinks et al. (2015) [108], aiming at T*b*NMT inhibition. Thereby, virtual screening based on the pharmacophore model was performed using 8773 approved drugs, and after visual inspection, 200 compounds were selected for purchase. Thus, *hit* compounds **51** and **52** (Figure 17) were optimized to provide compound **53** (Figure 17), with excellent results against *T. brucei* (IC_50_ = 0.27 µM, EC_50_ = 6.3 µM). Next, compound **52** yielded compound **54** (Figure 17) and six analogs, which could facilitate additional interactions and exhibited increased potency (IC_50_ values all under 0.010 µM). One of these analogs, compound **55** (Figure 17), exhibited potent antiparasitic activity in *T. brucei* cells (EC_50_ = 0.007 µM) and showed promising in vitro drug metabolism and pharmacokinetics profiles, demonstrating the potential for this scaffold to be further optimized as an oral antiparasitic drug.

## 5. Future Directions

Despite being old and well-known, NTDs continue to threaten the health of the world population, and control measures are necessary. The need for new therapies is evident due to the limitations of the current therapeutic arsenal, such as drug toxicity and high levels of resistance. Furthermore, identifying promising targets remains a significant challenge in the search for drugs against these diseases. Here, the importance of NMT in the search for new drugs against NTDs, particularly leishmaniasis and HAT, as well as related diseases such as malaria, was demonstrated. Interfering with the protein myristoylation process by inhibiting NMT may be a promising approach that could lead to a new antiparasitic mechanism of action. However, as shown here, target selectivity remains a significant challenge due to the high homology of parasitic and human NMTs. Ongoing studies are increasingly aiming to overcome this limitation, primarily with the aid of computational methods in the search for increasingly specific patterns of target selectivity. This is a critical aspect for developing drugs against parasitic diseases, as it helps avoid side effects. Furthermore, the human NMT inhibitor Zelenirstat is being evaluated in clinical anticancer trials, and this shows the importance of this drug target and its potential in drug development, and once again, the importance of target selectivity [109]. Some works highlight the importance of Tyr^211^ in improving the target selectivity against P*v*NMT, and this insight can be critical in designing new inhibitors. Therefore, identifying structural aspects that may increase selectivity against parasitic NMT is crucial to avoid off-target reactivity, which contributes to the clinical evolution of the compounds presented here. Furthermore, it is clear that the importance of pyrazole nuclei lies in the development of new NMT inhibitors. The scaffold provides critical insights, and the next step is to use this core to identify critical patterns of target selectivity. In addition, heterocyclic amines such as aminoacylpyrrolidine show their importance, and exploring the binding mode of new analogs is critical to discovering new NMT inhibitors more selectively.

Another limitation that needs to be overcome is the difficulty of studies *featuring* in vivo assays. Most of the studies presented here are based on in silico assays preceded by target-specific or cellular assays. However, to support the findings, there is still a need for in vivo assays that can confirm the compounds’ potential and search for a molecule that can be used in future clinical trials. These gaps still require support to ensure the development of compounds as a future hope against these diseases.

Although several NMT characteristics essential for drug design have been elucidated, many compounds have had no further studies carried out beyond in silico evaluation, providing no information on how these molecules behave when interacting with the isolated enzyme. Therefore, experimental validation of these findings is essential to continue future studies. In addition, CADD methods prove essential in discovering new drugs, revealing critical information that can be used further to optimize the development of innovative drugs against these diseases. However, these methods have limitations, including the restriction on target flexibility in molecular docking and the limitations of force fields in MD simulations in accurately estimating the electronic properties of atoms. Thus, the perspective is to improve these aspects of CADD methods to enhance the probability of identifying a critical clinical compound through them.

Finally, for the main compounds identified here as NMT inhibitors against leishmaniasis, malaria, and HAT, Table 1 highlights the most promising developments that can guide researchers in using CADD methods to discover new drugs against these diseases.

## 6. Conclusions

This work revealed critical information about the CADD methods used to discover new drugs against leishmaniasis, HAT, and malaria targeting NMT. In fact, the CADD methods are critical to discovering new drugs. Several works presented here highlight their importance in screening new compounds, increasing the probability of success in biological assays, and generating an efficient economy in terms of time and costs. Here, for those molecules that were indeed further studied through biological assays, some failed to inhibit the parasites, even though they had good performance in inhibiting the enzymes, exhibiting lower performance compared to market-available standard drugs. As highlighted in some works, this may be due to the membrane permeability of the compounds, which should be improved in future perspectives. Finally, a significant number of compounds were not tested in cell cultures or animal models, while some that were tested showed poor pharmacokinetics. Therefore, to advance the inhibitors discussed above into novel antiparasitic drugs, further analysis of their inhibitory and pharmacokinetic properties is warranted through both in vitro and in vivo assays so that the most promising compounds may be optimized and sent to clinical trials. In addition, scaffolds based on sulfones and pyrazoles are promising against T*b*NMT and L*m*NMT, while quinolines and benzothiophene are promising against P*v*NMT and P*f*NMT. Exploring these compounds in search of improved activity and target selectivity can lead to the development of new drugs with innovative mechanisms of action that can combat these threatening agents. Furthermore, the selectivity and improvement in activity are related to interactions with the residues Val^81^, Phe^90^, Tyr^217^, Tyr^326^, Tyr^345^, and Met^420^ for leishmaniasis, Tyr^211^, Leu^410^, and Ser^319^ for malaria, and Lys^25^ and Lys^389^ for HAT. These data can help researchers worldwide discover innovative drugs targeting NMT against several NTDs and mitigate the threat posed by these diseases.

## Figures and Tables

**Table 1:** Summary of the main compounds identified here as potential NMT inhibitors against leishmaniasis, malaria, and HAT using CADD approaches.

N°	Proposed Disease	Activity Value	Ref.	N°	Proposed Disease	Activity Value	Ref.
**(1)**	Leishmaniasis	*Lm*NMT: K*_i_* = 250 nM	[86]	**(29)**	Malaria	-	[98]
**(2)**	Leishmaniasis	*Lm*NMT: K*_i_* = 248 nM	[86]	**(30)**	Malaria	-	[98]
**(3)**	Leishmaniasis	*Lm*NMT: K*_i_* = 250.86 nM	[86]	**(31)**	Malaria	-	[98]
**(4)**	Leishmaniasis	*Lm*NMT: K*_i_* = 235.74 nM	[86]	**(32)**	Malaria	*Pf*NMT: K_i_ = 832 nM *Pv*NMT: K_i_ = 84 nM	[81]
**(5)**	Leishmaniasis	-	[86]	**(33)**	Malaria	*Pf*NMT: K_i_ = 8 nM *Pv*NMT: K_i_ = 2 nM *P. falciparum*: EC_50_ = 302 nM	[81]
**(6)**	Leishmaniasis	*Lm*NMT: K*_i_* = 912.48 nM	[86]	**(34)**	Malaria	*Pv*NMT: K_i_ = 4.7 µM *Pf*NMT: K_i_ = >100 µM	[78]
**(7)**	Leishmaniasis	*Ld*NMT: IC_50_ = 0.024 ± 0.003 µM *Hs*NMT: IC_50_ = 0.06 ± 0.003 µM	[88]	**(35)**	Malaria	*Pv*NMT: K_i_ = 0.44 µM *Pf*NMT: K_i_ = 0.67 µM	[78]
**(8)**	Leishmaniasis	*Ld*NMT: IC_50_ = 0.482 µM	[80]	**(36)**	Malaria	*Pv*NMT: K_i_ = 0.34 µM *Pf*NMT: K_i_ = 0.96 µM	[78]
**(9)**	Leishmaniasis	*Lm*NMT: IC_50_ = 0.318 µM	[80]	**(37)**	Malaria	*Pv*NMT: K_i_ = 0.34 µM *Pf*NMT: K_i_ = 0.96 µM *Pf*3D7: IC_50_ = 3.96 µM *Pf*INDO: IC_50_ = 6.38 µM	[78] and [102]
**(10)**	Leishmaniasis	*Ld*NMT: IC_50_ = 0.077 µM	[80]	**(38)**	Malaria	*Pf*3D7: IC_50_ = 6.71 µM *Pf*INDO: IC_50_ = 2.8 µM	[102]
**(11)**	Leishmaniasis	*Ld*NMT: IC_50_ = 0.158 µM	[80]	**(39)**	Malaria	*Pf*NMT: pIC_50_ = 7.301	[103]
**(12)**	Leishmaniasis	*Ld*NMT: K_i_ = 100 nM *L. donovani*: EC_50_ > 50 µM (amastigotes)	[90]	**(40)**	Malaria	*Pf*NMT: pIC_50_ = 7.014	[103]
**(13)**	Leishmaniasis	*Ld*NMT: K_i_ = 1.6 nM *L. donovani*: EC_50_ = 10–30 µM (amastigotes)	[90]	**(41)**	Malaria	*Pf*NMT: pIC_50_ = 7.171	[103]
**(14)**	Leishmaniasis	Docking score: −13.93 kcal/mol Binding energy: −102.46 kcal/mol	[91]	**(42)**	Malaria	*Pf*NMT: pIC_50_ = 7.622	[103]
**(15)**	Leishmaniasis	Docking score: −14.75 kcal/mol Binding energy: −83.01 kcal/mol	[91]	**(43)**	Malaria	*Pf*NMT: pIC_50_ = 8.161	[103]
**(16)**	Leishmaniasis	GRID score: −63.87 kcal/mol	[92]	**(44)**	Malaria	*Pf*NMT: pIC_50_ = 7.894	[103]
**(17)**	Leishmaniasis	Binding affinity (docking): −22.2 kcal/mol	[93]	**(45)**	HAT	*Tb*NMT: QSAR Analysis	[104]
**(18)**	Leishmaniasis	Binding affinity (docking): −23.3 kcal/mol	[93]	**(46)**	HAT	*Tb*NMT: QSAR Analysis	[104]
**(19)**	Leishmaniasis	Binding affinity (docking): −24.0 kcal/mol	[93]	**(47)**	HAT	*Tb*NMT: QSAR Analysis	[104]
**(20)**	Malaria	-	[96]	**(48)**	HAT Filariasis	*Tb*NMT: QSAR Analysis *Ce*NMT: IC_50_ = 10 nM *Bm*NMT: IC_50_ = 10 nM	[104] and [110]
**(21)**	Malaria	-	[96]	**(49)**	HAT	*Tb*NMT (predicted): pIC_50_ = 8.70	[107]
**(22)**	Malaria	*Pv*NMT: IC_50_ = 80.15 nM	[96]	**(50)**	HAT	*Tb*NMT (predicted): pIC_50_ = 7.52	[107]
**(23)**	Malaria	*Pv*NMT: IC_50_ = 9.48 nM *P. vivax*: EC_50_ = 2.3–4.6 µM (schizonts) EC_50_ = 1.7 µM (hypnozoites)	[96]	**(51)**	HAT	*Tb*NMT: IC_50_ = 22 µM *T. brucei*: EC_50_ > 50 µM	[108]
**(24)**	Malaria	*Pf*NMT: K_i_ = 1.4 µM	[97]	**(52)**	HAT	*Tb*NMT: IC_50_ = 12 µM *T. brucei*: EC_50_ > 50 µM	[108]
**(25)**	Malaria	*Pf*NMT: K_i_ = 1.6 µM	[97]	**(53)**	HAT	*T. brucei*: IC_50_ = 0.27 µM EC_50_ = 6.3 µM	[108]
**(26)**	Malaria	PfNMT: K_i_ = 0.95 µM	[97]	**(54)**	HAT	*Tb*NMT: IC_50_ = 2.9 µM	[108]
**(27)**	Malaria	*Pv*NMT: K_i_ = 0.027 µM	[97]	**(55)**	HAT	*Tb*NMT: IC_50_ < 0.002 µM *T. brucei*: EC_50_ = 0.007 µM	[108]
**(28)**	Malaria	*Pf*NMT: K_i_ = 0.0017 µM	[97]				

## Data Availability

Not applicable.

## Abbreviations

ACTs	Artemisinin-Based Combination Therapies
ADMET	Absorption, Distribution, Metabolism, Excretion, and Toxicity
AI	Artificial Intelligence
CADD	Computer-Aided Drug Design
CD	Chagas Disease
CNS	Central Nervous System
DALYs	Disability-Adjusted Life Years
HAT	Human African Trypanosomiasis
H*s*NMT	*Homo sapiens N*-myristoyltransferase
LBDD	Ligand-Based Drug Design
*Ld*NMT	*Leishmania donovani N*-myristoyltransferase
LELP	Ligand Efficiency-Dependent Lipophilicity
L*m*NMT	*Leishmania major N*-myristoyltransferase
LPG	Lipophosphoglycan
MCL	Mucocutaneous Leishmaniasis
MyrCoA	Myristoyl-CoA
NMT	*N*-myristoyltransferase
NPOs	Non-Profit Organizations
NTDs	Neglected Tropical Diseases
PDB	Protein Data Bank
*Pf*NMT	*Plasmodium falciparum N*-myristoyltransferase
P*v*NMT	*Plasmodium vivax N*-myristoyltransferase
QSAR	Quantitative Structure–Activity Relationship
R_g_	Radius of Gyration
RMSD	Root Mean Square Deviation
RMSF	Root Mean Square Fluctuation
ROCs	Receiver Operating Characteristic
SBDD	Structure-Based Drug Design
SI	Selectivity Index
SSG	Sodium Stibogluconate
T*b*NMT	*Trypanosoma brucei N*-myristoyltransferase
VL	Visceral Leishmaniasis
VSAs	Variant Surface Antigens
WHO	World Health Organization
